# New genera, species and records of Phaneropterinae (Orthoptera, Phaneropteridae) from sub-Saharan Africa

**DOI:** 10.3897/zookeys.472.8575

**Published:** 2015-01-19

**Authors:** Bruno Massa

**Affiliations:** 1 Department of Agricultural and Forest Sciences, University of Palermo, V.le Scienze 13, 90128 Palermo, Italy

**Keywords:** Distribution, taxonomy, revision, tropical Africa

## Abstract

The results of the study of many specimens preserved in different European museums are reported. The tribe Terpnistrini Brunner von Wattenwyl, 1878 is resurrected. The distribution of the following species is enhanced: *Pardalota asymmetrica* Karsch, 1896, *Diogena denticulata* Chopard, 1954, *Diogena fausta* (Burmeister, 1838), *Plangiopsis adeps* Karsch, 1896, *Poreuomena sanghensis* Massa, 2013 and *Tylopsis continua* (Walker, 1869). Further, for their peculiar characteristics, two African representatives of the American genus *Symmetropleura* Brunner von Wattenwyl, 1878 are included in two new genera: *Symmetrokarschia africana* (Brunner von Wattenwyl, 1878), **comb. n.** and *Symmetroraggea dirempta* (Karsch, 1889), **comb. n.** A new genus and species from the Democratic Republic of Congo, *Angustithorax spiniger* **gen. n., sp. n.**, and a new genus and species from Tanzania, *Arostratum oblitum* **gen. n., sp. n.** are described. Finally *Melidia claudiae* **sp. n.** and *Atlasacris brevipennis* **sp. n.** are described and compared with related species.

## Introduction

Central Africa is one of the richest areas of the world for Orthoptera. Despite the many studies carried out since 1800, this wide geographic region still hides many unknown taxa. One of the groups that shows an amazing diversity of forms and species is that of Phaneropteridae (sensu Heller et al. 2014). There are areas of tropical forests or high mountains where many species belonging to the same genus live together, and localities are known from which dozens of types of species come (e.g., Barombi Station in Cameroon).

The present paper is the result of the study carried out on material collected in sub-Saharan Africa by different collectors and preserved in various museums, and follows two other papers on the same subject (Massa 2013, 2014). It demonstrates that we still lack data on many interesting taxa, and very probably many others are waiting to be discovered.

## Material and methods

The study of series of sub-saharan African specimens was possible during visits to the following museums: Museo Nacional de Ciencias Naturales, Madrid; Museum für Naturkunde, Berlin; Museo Civico di Storia Naturale ‘G.Doria’, Genoa; Museo di Storia Naturale, University of Pavia; in addition some unidentified specimens from Democratic Republic of Congo were obtained on loan from the Museo di Storia Naturale of Terrasini (Palermo).

Abbreviations used in this paper:

BMCP Bruno Massa Collection, University of Palermo;

MfN Museum für Naturkunde, Berlin;

MNCN Museo Nacional de Ciencias Naturales, Madrid;

MRT Museo Regionale di Storia Naturale, Terrasini (Palermo);

MSNG Museo Civico di Storia Naturale ‘G.Doria’, Genoa;

MSNP Museo Storia Naturale, University of Pavia.

Some specimens were photographed with a Nikon Coolpix 4500 digital camera, mounted on a Wild M5 Stereomicroscope or Leika MZ75, and photos were integrated using the freeware CombineZP (Hadley 2008). Mounted specimens were measured with a digital calliper (precision 0.01 mm); the following measures were taken (all measurements in mm): body length: dorsal length from the head to the apex of the abdomen, ovipositor excluded in females; pronotum length: length of the pronotum along dorsal median line; pronotum height: maximum height of the pronotum; hind femur: length of hind femur; hind tibia: length of hind tibiae; tegmina: length of tegmina; ovipositor: maximum length.

## Results

### Tribe Pardalotini Brunner von Wattenwyl, 1878

#### Pardalota asymmetrica

Taxon classificationAnimaliaOrthopteraPhaneropteridae

Karsch, 1896

##### Material examined.

Democratic Republic of Congo, Nulunau (1800 m) 24.V.1970, T. De Stefani (♀) (MRT).

##### Distribution.

*Pardalota asymmetrica* was described by Karsch (1896) on a series of specimens from Uganda and Tanzania (see also Hollier 2010). It has been recently recorded in the Democratic Republic of Congo by Heller et al. (2014). This new record extends the known distribution of this species westwards.

### Tribe Terpnistrini Brunner von Wattenwyl, 1878

**Figures 1–6.**
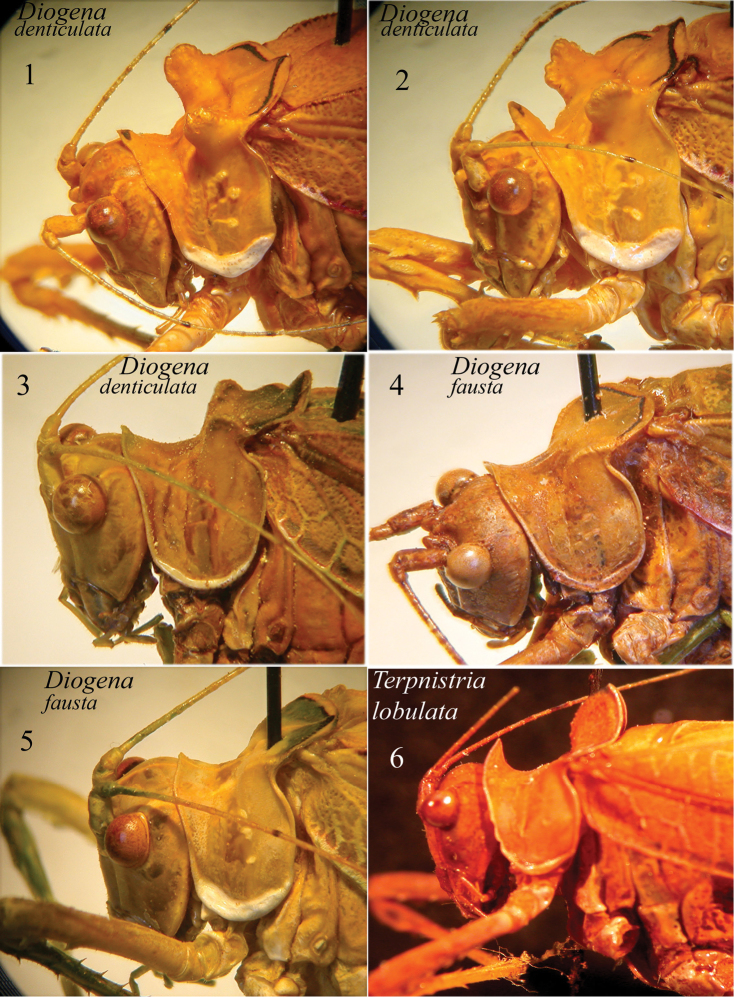
Tribe Terpnistrini. **1–3** *Diogena denticulata* from Bud Bud (**1**), El Da (**2**) and Mogadishu (Somalia) (**3**) **4–5** *Diogena fausta* from Burkina Faso **6** *Terpnistria lobulata* from Namibia.

**Remarks.** Following Ragge (1980) *Terpnistria* Stål, 1873, *Diogena* Brunner von Wattenwyl, 1878 (Figs 1–6) and *Tropidophrys* Karsch, 1896 are most unusual among African Phaneropterinae; the dorsal spurs of the fore and mid tibiae are usually replaced towards the base by broad-based spines; in *Gelotopoia* Brunner von Wattenwyl, 1891 the fore and mid tibiae have no dorsal spurs except at the apex, but the mid tibiae have broad-based, thorn-like dorsal spines on each side. *Terpnistria*, *Diogena* and *Tropidophrys* form a distinct group with thorn-like spines on the legs, frontogenal carinae on the head and characteristic coloration. The fact that they are allopatric suggests a comparatively recent divergence from a more widespread ancestor. *Terpnistrioides* Ragge, 1980 and *Gelotopoia* also seem to stand somewhat apart from the other three genera of the group. Altogether, their general habitus is rather similar, mainly in the shape of the pronotum and the external genitalia. They are generally treated together as a genus group, but the tribe Terpnistrini should be resurrected for these five genera, which is proposed in this study.

#### Diogena denticulata

Taxon classificationAnimaliaOrthopteraPhaneropteridae

Chopard, 1954

Figs 1 , 2 , 3

##### Material examined.

Somalia, Mogadishu 1937, A.Negrotto, I.Cambiasol (♀); Somalia, Bud Bud 28–30.XI-4.XII.1982, S.B.S. (♀); Somalia, El Da 6–7.XII.1982, S.B.S. (♀) (MSNG).

##### Remarks.

According to Chopard (1954), the anterior margin of pronotum is elevated in a triangular point, posterior margin is acute in the middle, lateral tubercles are massive, a little compressed at their upper extremities, irregularly truncated, the female sub-genital plate is triangular, a little rounded at the apex, and foliaceous spines of the hind femora are much wider than in *Diogena fausta*, the last two contiguous. The smallest in size of the above specimens has not the triangular point on the fore margin of the pronotum and lateral tubercles are much variable (Figs 1–5).

##### Distribution.

*Diogena denticulata* was described from two females from El Wak (Kenya), not far from the border with Somalia. The male is still unknown. Localities of Somalia listed above are northeast of Mogadishu and next to the border with Kenya; thus, this species has a wider distribution in East Africa and is not confined to Kenya and Tanzania (Hemp 2013).

#### Diogena fausta

Taxon classificationAnimaliaOrthopteraPhaneropteridae

(Burmeister, 1838)

Figs 4 , 5

##### Material examined.

Burkina Faso, Pama VIII.2004, P.Moretto (♀); Burkina Faso, Yako VIII.2005, P.Moretto (♂); Burkina Faso, Gorom Gorom, Essakane 10–13.IX.2012, 22–24.VIII.2012, 2–4.X.2012, 14.X.2012 (UV trap), P.Moretto (6♂, 9♀); Burkina-Faso, Boromo, Ft. of Sorobouli 4–5.VII.2013 (UV trap), P.Moretto (1♂, 1♀); Burkina-Faso, Dori 23–27.VIII.2013, 30.VIII–5.IX.2013 (UV trap), P.Moretto (6♂, 3♀); Senegal, Niokolo Korba VIII.2008, P.Moretto (♀); Senegal, Niolo du Rip VII.2008, P.Moretto (♂); Guinea, Ziama Forest near Seredou 7.VII.2004, A.Kudrna (♀); Central African Republic, Dzanga-Ndoki National Park, Lac 3, 25–26.II.2012 (UV trap), P.Moretto (♀) (BMCP); Democratic Republic of Congo, Yangambi IV.1964, M.Pavan (2♀) (MSNP).

##### Distribution.

*Diogena fausta* is known from a wide area covering the Middle East, North Africa, from Egypt to Mauritania, the Arabian Peninsula, semi-desert regions of south of the Sahara, East Africa and West Africa (Senegal, the Ivory Coast); the presence in the Central African Republic and Burkina Faso was hitherto unknown (Chopard and Mc Kevan 1954, Ragge 1968a, 1980, Popov 1981).

### Tribe Phaneropterini Burmeister, 1838

#### Genus Symmetropleura

Taxon classificationAnimaliaOrthopteraPhaneropteridae

Brunner von Wattenwyl, 1878

##### Remarks.

When Brunner von Wattenwyl (1878) described the genus *Symmetropleura* (from Latin: *symmetro* = symmetric, equal; *pleura* = side), he placed it in the American group of Scudderiae. According to Brunner von Wattenwyl (1878) characters of the genus are the following: fastigium of vertex triangular and sulcate; pronotum disc flat, with lateral excisions, anterior margin straight, and rounded posterior margin; tegmina wide with rounded hind margin or narrow with straight hind margin; fore and mid femora with ventral inner spines, hind femora with double row of ventral spines. Fore and mid tibiae dorsally unarmed or with some spinules; cerci long, in-curved and pointed; male sub-genital plate short with rounded posterior margin or (in *Symmetropleura africana*) long with triangular apex; styli absent; ovipositor longer than pronotum, not much curved, sharp, with upper and lower apices serrate (differently shaped in *Symmetropleura africana*: see below); female sub-genital plate triangular, just concave. In the description Brunner von Wattenwyl (1878) referred mainly to *Symmetropleura laevicauda*, both within the text and in the figure 73; thus, by subsequent designation, Kirby (1906) established *Symmetropleura laevicauda* as the type-species of the genus. The description of the female of *Symmetropleura laevicauda* by Brunner von Wattenwyl (1878) from Bahia (Brasil) is as follows (translated from Latin): small (28 mm), tegmina width about two times the length of pronotum, anterior margin basally pale with a darker outer area, posterior margin rounded. Radius forked before Media, ovipositor little up-curved, acuminate.

Ragge (1968b, 1980) pointed out that *Symmetropleura* is a New World genus, occurring in South America, Mexico and eastern USA, and that the two African species are not very similar either to each other or to the Neotropical type-species of the genus. He observed also that the name *Cameronia* Karsch, 1888 was available for *Symmetropleura africana*. However, the genus *Cameronia* should be considered a junior homonym, because was pre-occupied since 1879, when *Cameronia spekii* Bourguignat (Mollusca Bivalvia) was described from Lake Tanganyika (R. Poggi, pers. comm.)^1^.

There are three African species so far included in the genus *Symmetropleura*: one of them is *Symmetropleura africana*, others are *Symmetropleura dirempta* Karsch, 1889, that occurs in Madagascar, treated below, and *Symmetropleura plana* (Walker, 1869), that occurs in South Africa. Concerning the latter, some photographs, kindly taken by C. Hemp, show that the pronotum is not keeled and the male sub-genital plate has a very different shape from that of *Symmetropleura africana* and *Symmetropleura dirempta*; it is very probable that it belongs to another undescribed genus, but specimens were not available to establish this.

For the reasons reported above and below, two new genera are described for *Symmetropleura africana* and *Symmetropleura dirempta*.

#### Genus Symmetrokarschia gen. n.

Taxon classificationAnimaliaOrthopteraPhaneropteridae

http://zoobank.org/DE10724C-FD67-483D-9611-B663BD047F0D

##### Type-species.

*Symmetropleura africana* Brunner von Wattenwyl, 1878, here designated.

The original description of the male holotype of *Symmetropleura africana* Brunner von Wattenwyl, 1878, from Congo is the following (translated from Latin): large (37 mm), pronotum disc with regular impressed punctures, wide tegmina, with rounded hind border, radius forked before media, male tenth tergite laminate and protruding, with straight hind border, cerci little in-curved, with flat apex and pointed, sub-genital plate long, narrow, with obtuse and short cut apex; the description of the female from Chinchoxo (Cameroon) by Karsch (1889) reports the ovipositor shape, shorter than pronotum, with upper border and apex of lower border finely serrulate. Karsch (1889) observed the differences between *Symmetropleura africana* and others of the genus and reported it as: Symmetropleura (Cameronia) africana. Later, only Bolívar (1906) recorded this species with the name used by Karsch, which we hardly may interpret as a subgenus, not still used at that time (cf. Ragge 1968b).

##### Etymology.

After the German entomologist and arachnologist Ferdinand Karsch (1853–1936), whose contribution to the knowledge of tropical African Orthoptera was really remarkable; he also observed the differences between African and American species of the genus *Symmetropleura*.

##### Remarks.

Main differences between *Symmetrokarschia* and *Symmetropleura* are: in *Symmetropleura* lateral margins of metanotum are keeled, male sub-genital plate is short with rounded margin, ovipositor is longer, basally straight and gently arcuate in the posterior part.

#### Symmetrokarschia africana

Taxon classificationAnimaliaOrthopteraPhaneropteridae

(Brunner von Wattenwyl, 1878) comb. n.

Figs 7–13

##### Material examined.

Cameroon, Chinchoxo (2♀) (MfN); Angola, Dundo (1♂) (MCNM).

##### Redescription.

**Figures 7–13.**
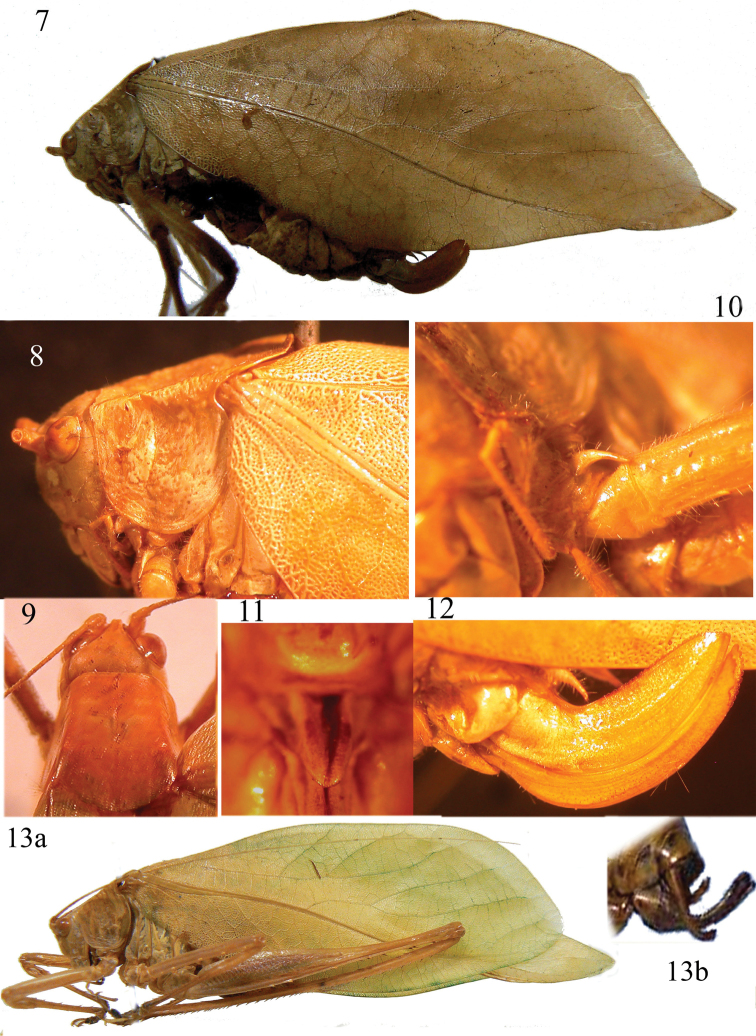
*Symmetrokarschia africana* (Brunner von Wattenwyl, 1878) from Cameroon. Lateral view of female (**7**), pronotum in lateral view (**8**) and from above (**9**), spine on fore coxa (**10**), sub-genital plate of female ovipositor (**11**), lateral view of the ovipositor (**12**), lateral view of male (**13a**) and last abdominal segments of male (**13b**).

Fastigium of vertex compressed, narrower than first antennal segment, sulcate above (Fig. 9). Eyes oval, prominent, without fronto-genal carinae. Pronotum just depressed, fore part with just definite lateral carinae, central and hind parts with vague lateral carinae; fore margin slightly concave, posterior margin rounded; surface dotted, matt (Figs 7–9). Fore coxae with a long spine (Fig. 10). Fore tibiae with open tympanum on each side, furrowed on upper border. Fore and mid femora with 3–5 spines, hind femora with 5–8 inner ventral and 6–7 outer ventral spines. Fore and mid tibiae with 1 dorsal and 1 ventral spur, hind tibiae with 3 apical spurs on each side. Male tenth tergite laminate and protruding, with straight posterior margin, cerci little in-curved, with flat apex and pointed, sub-genital plate long, narrow, with obtuse and short cut apex, styli absent (Fig. 13b). Ovipositor well developed, sharply bent upwards near the base, shorter than pronotum, with upper border and apex of lower border finely serrate (Fig. 12), sub-genital plate triangular and pointed (Fig. 11). Tegmina are oval, more pronounced in the female (Fig. 7) than in male (Fig. 13a).

##### Distribution.

Democratic Republic of Congo, Cameroon and Angola.

#### Genus Symmetroraggea gen. n.

Taxon classificationAnimaliaOrthopteraPhaneropteridae

http://zoobank.org/DC6C78C5-B2E5-402E-A694-B847E654CFFC

##### Type-species.

*Symmetropleura dirempta* Karsch, 1889, here designated.

Among the species described within the genus *Symmetropleura* Brunner von Wattenwyl, 1878, Karsch (1889) included also *Symmetropleura dirempta* from Madagascar, that has the characters reported below, not corresponding to those of the genus *Symmetropleura* (see above the translation of the description of the type-species *Symmetropleura laevicauda*, in particular the round margin of upper tibiae and the fronto-genal carinae present in *Symmetroraggea* gen. n.).

##### Etymology.

The genus is dedicated to David R. Ragge, authority on African Phaneropteridae taxonomy, who also pointed out the differences of African *Symmetropleura* when compared to American species (Ragge 1968b, 1980).

#### Symmetroraggea dirempta

Taxon classificationAnimaliaOrthopteraPhaneropteridae

(Karsch, 1889) comb. n.

Figs 14–19

##### Material examined.

Madagascar, Nossi bé, Hildabrandt (♂ holotype of *Symmetroraggea dirempta*) (MfN); it bears a label with a former identification: *laevicauda* Brunner.

##### Redescription.

**Figures 14–19.**
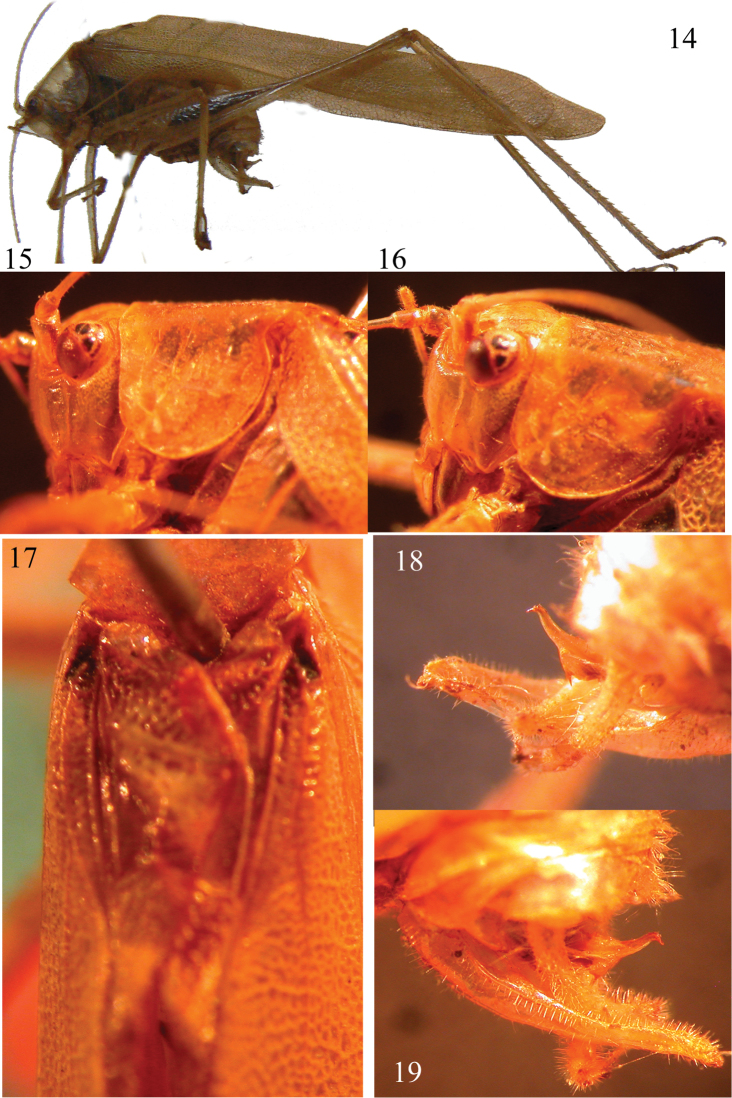
*Symmetroraggea dirempta* (Karsch, 1888), male holotype from Madagascar. Lateral view of habitus (**14**), lateral view of head and pronotum (**15**), lateral view of head (**16**), dorsal view of tegmina (**17**), right (**18**) and left (**19**) view of genitalia.

Eyes oval, with fronto-genal carinae below them (Figs 15–16); fastigium clearly narrower than first antennal segment, sulcate above. Flat pronotum, without lateral carinae, with the exception of the last part of metanotum, whose margins are sharp (Figs 15–16). Very narrow tegmina, with posterior margin nearly straight (Fig. 14), Radius of wing just forked before media, the fore base is black, as well as a longitudinal line bordering the stridulatory area and extending posteriorly with a wide marking on left tegmen (Fig. 17); coxae armed, 6 spines on ventral margin of fore femora, 6 inner spines plus 1 spur and 6 outer spines plus 1 spur on ventral margins of fore tibiae, fore tibiae dorsally rounded, not sulcate, mid femora with 7 outer spines, mid tibiae with 12 outer spines plus 1 spur and 5 inner spines plus 1 spur, hind femora with 7 outer and 4 inner spines, hind tibiae with 13 outer and 11 inner spines plus 3 spurs in each side; male tenth tergite posteriorly not protruding, with hind margin concave, male supra-genital plate protruding, with very acuminate apex, cerci stout, long, in-curved, with pointed apex, male sub-genital plate exceeding cerci, long, triangular, posteriorly narrowed, with deeply cut apex, whose margins are parallel and close between them (Figs 18–19); titillators^2^ are present and show a long, downcurved and hooked apex, with 2 spines basally and 3–4 small spines dorsally on each border (Figs 18–19).

##### Distribution.

*Symmetroraggea* gen. n. is known only from Madagascar, where only one species has been recorded, *Symmetroraggea dirempta* (Krauss, 1889), of which only the type is known.

#### Plangiopsis adeps

Taxon classificationAnimaliaOrthopteraPhaneropteridae

Karsch, 1896

##### Material examined.

Cameroon, Lolodorf, L.Conradt (2♀ syntypes) (MfN); Central African Republic, Dzanga-Ndoki National Park, Ndoki, Lake 1 10–11.II.2012 (UV trap), P.Moretto (1♀); same data 28–29.II.2012 (1♂); same data 29.II–1.III.2012 (1♀); Ivory Coast, Bondoukou Zamou VII.2004, P.Moretto (2♂, 4♀); Ivory Coast, Sassandra I.1998, P.Moretto (1♂); Togo, Fazao hotel 3–4.VIII.2013 (UV trap), P.Moretto (2♀); Togo, Kpalimé, Ft of Missahöhe 29–30.VII.2013, P.Moretto (1♂) (BMCP).

##### Remarks.

*Plangiopsis adeps* is the largest species of the genus; only females from Cameroon (Lolodorf) were described by Karsch (1896). New distribution data now suggest that this species is widespread throughout Cameroon and Central African Republic to the Ivory Coast and Togo. Sexual characters of the male, previously undescribed, are the following: cerci slender and long, in-curved and a little up-curved, sub-genital plate narrow and long, ending with a v-shaped concavity, styli long. The stridulatory file is comparatively long.

#### Poreuomena sanghensis

Taxon classificationAnimaliaOrthopteraPhaneropteridae

Massa, 2013

##### Material examined.

Cameroon, Bare-Dschang 2–6.XII.1908, Riggenbach (2♂); Cameroon, Victoria (3♀) (MfN).

Among specimens of *Poreuomena* of MfN there are 5 unidentified specimens of this species, recently described from Central African Republic (Massa 2013).

#### Genus Melidia

Taxon classificationAnimaliaOrthopteraPhaneropteridae

Stål, 1876

Figs 20 –31

##### Remarks.

The genus *Melidia* Stål, 1876 is characterized by the fastigium of the vertex, which is prominently raised in the region of the lateral ocelli. It looks like the genus *Phaneroptera*, but differs from this mainly in the shape of the head, the male cerci and the sub-genital plate, and broader fore wings. It has a well-developed spine on the fore coxae and males have the stridulatory region of the left tegmina brown or conspicuously marked with brown (Ragge 1980).

**Figures 20–25.**
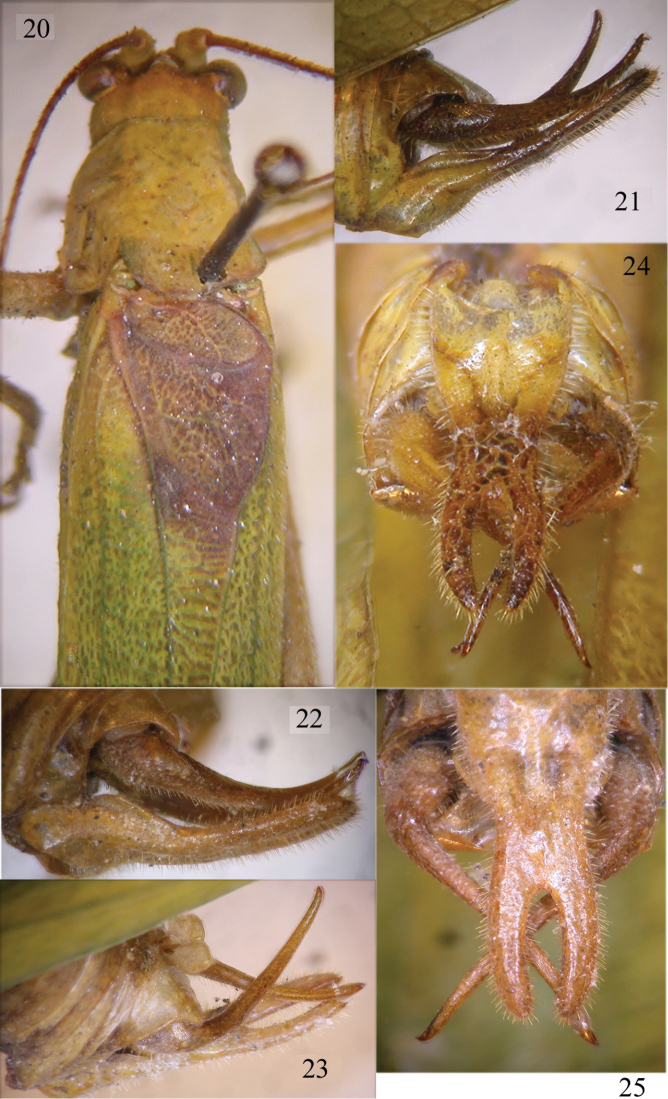
*Melidia claudiae* sp. n. Head, pronotum and tegmina of holotype from above (**20**), lateral view of cerci and sub-genital plate of holotype (**21–22**) and paratype (**23**), ventral view of the sub-genital plate of the holotype (**24**) and paratype (**25**).

**Figures 26–31.**
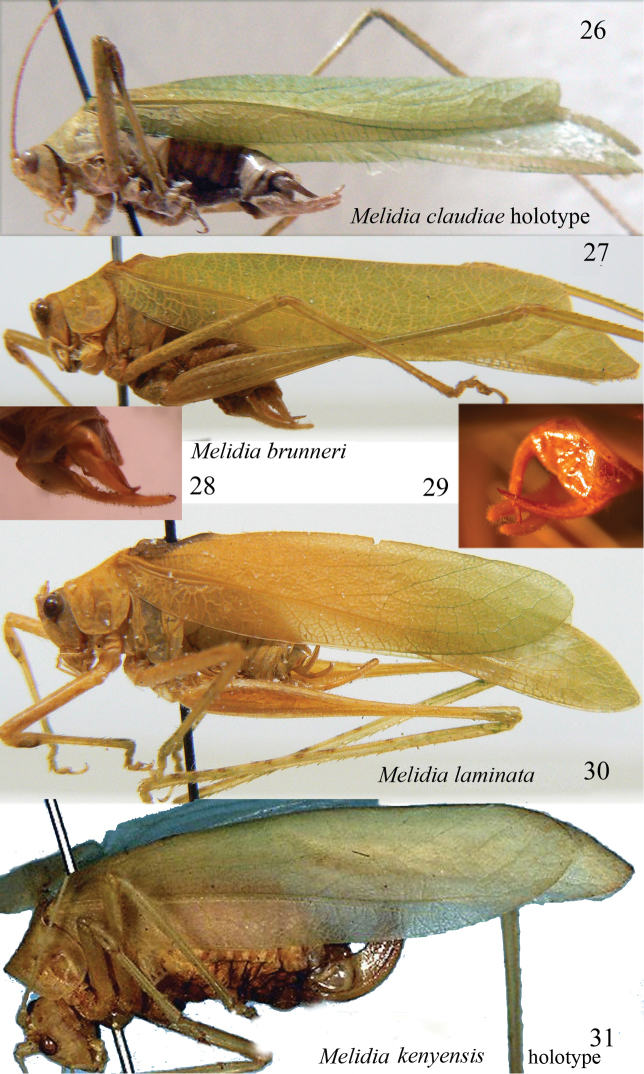
Species of the genus *Melidia*. Lateral view of *Melidia claudiae* sp. n. (**26**), lateral view of *Melidia brunneri* holotype (**27**), cerci and sub-genital plate of *Melidia brunneri* from Namibia (**28**), cerci and sub-genital plate of *Melidia laminata* (**29**), lateral view of *Melidia laminata* (**30**) and *Melidia kenyensis* (**31**). Photo **27**, **29**, **30** and **31** by S. Ingrisch for OSF.

#### Melidia claudiae sp. n.

Taxon classificationAnimaliaOrthopteraPhaneropteridae

http://zoobank.org/90BC4041-BD4F-4B19-A729-D66AFD4FE46B

Figs 20 –26

##### Material examined and depository.

*Melidia claudiae* sp. n.: Democratic Republic of Congo, Lubumbashi (11°42'1.06"S, 27°31'57.07"E) 1.II.1968 (♂ holotype), 5.II.1968 (♂ paratype), T. De Stefani (MRT). *Melidia laminata* Chopard, 1954: Tanzania, Kilimanjaro (♂) (MCNM). *Melidia brunneri* Stål, 1876: Namibia (♂) (MCNM); Namibia, Okahandja (4♂, 2♀); Namibia, Okahandja, near Waterberg 1936, W.Hoesch (1♂, 2♀); Namibia, Gobabis I.1897 (1♂); Namibia, Keetmanshoop (1♂); Namibia (1♂); SW Africa 11.XI.1903 (1♂); South Africa, Rietfontein II.1897, Borchmann (1♂) (MfN). *Melidia kenyensis* Chopard, 1954: Kenya (♀ holotype in OSF).

##### General habitus and colour.

Yellow with green tegmina, antennae brownish. The stridulatory region of left tegmen is brown. Abdomen yellowish with brown vertical stripes on posterior margins of tergites.

##### Description.

Male. Medium sized (Fig. 26). Head and antennae: fastigium of vertex very narrow, scarcely furrowed above, separated from the fastigium of frons, which is tuberculated. Eyes rounded, well projecting (Fig. 20). Legs comparatively long, green. Fore coxae armed with a well-developed spine. Fore tibiae furrowed on upper margin, distinctly widening above tympanum, which is open on inner and outer sides. Fore femora armed on inner ventral margin with 3–4 spines, fore tibiae with 1 spine plus 1 spur on inner and outer ventral margins, 1 spur on outer dorsal margin, mid femora armed with 2 spines on outer ventral margin, mid tibiae with 8 on outer and 5 spines on inner ventral margins, plus 1 spur on each side, hind femora armed with 3–4 small spines on outer and 2 on inner ventral margins, hind tibiae with many spines on ventral and dorsal margins and 3 spurs on each side. Thorax: pronotum little narrowing anteriorly, flat above, anterior margin straight, posterior margin rounded, humeral sinus evident, lobes of pronotum rounded (Fig. 20). Tegmina comparatively narrow with convex fore margins and rounded apices. Wings longer than tegmina (Fig. 26). The stridulatory region of left tegmen is long (Fig. 20). Abdomen: tenth tergite with a straight hind margin; sub-genital plate long and deeply divided into two robust in-curved lobes; styli absent (Figs 24–25). Cerci long, fine and decussate, longer than the sub-genital plate (Figs 21–23).

Female. Unknown.

##### Etymology.

*Melidia claudiae* is dedicated to the German orthopterist Claudia Hemp, who is working with competence and great interest on the Orthoptera of tropical Africa, Phaneropteridae in particular.

##### Distribution.

Democratic Republic of Congo. Considering that other species are currently known from Kenya, Tanzania, Nigeria, Botswana, South Africa and Namibia (Chopard 1954, Ragge 1980, Hemp 2013), it is likely that the genus covers a wider distribution and further species will be found.

##### Measurements.

Body length: 15.1–15.2; pronotum length: 3.6–3.9; pronotum height: 3.0–3.2; hind femur: 17.0–17.5; tegmina: 25.9–26.5.

##### Diagnosis.

*Melidia kenyensis* Chopard, 1954 was described from Kenya (Chopard 1954). Although only the female is known, it has a different shape of tegmina than *Melidia claudiae* sp. n. (Fig. 31). *Melidia laminata* Chopard, 1954 (Fig. 30), described from Kenya (Wajir, El Katulo) and recorded from Kilimanjaro (Tanzania) by Hemp (2013) is more similar in tegmina shape to the new species, but the male sub-genital plate is longer, surpassing the cerci (Fig. 29). *Melidia brunneri* Stål, 1876 (Fig. 27) from southern and central Africa (Ragge 1980) is more similar to *Melidia claudiae* sp. n., but differs from it in the shape of the male sub-genital plate, which is longer than cerci (Fig. 28). Further, the cerci of *Melidia brunneri* are more robust and shorter than those of *Melidia claudiae* sp. n. (compare Figs 23 and 28), and the tegmina are broader than those of *Melidia claudiae* sp. n. (compare Figs 26 and 27).

#### Tylopsis continua

Taxon classificationAnimaliaOrthopteraPhaneropteridae

(Walker, 1869)

##### Material examined.

Democratic Republic of Congo, Goma 14.I.1968, T. De Stefani (♂, morph with unicolor pronotum) (MRT).

##### Distribution.

According to Ragge (1964), *Tylopsis continua* is widespread in southern Africa, with records also in Zimbabwe, Mozambique, Tanzania and Angola. The record from Goma, on the border between the Democratic Republic of Congo and Rwanda, expands the distribution of this species further.

#### Genus Angustithorax gen. n.

Taxon classificationAnimaliaOrthopteraPhaneropteridae

http://zoobank.org/24EA627C-9066-466F-9B2C-08426273483E

##### Type species.

*Angustithorax spiniger* sp. n., here designated.

##### Description.

**Figures 32–38.**
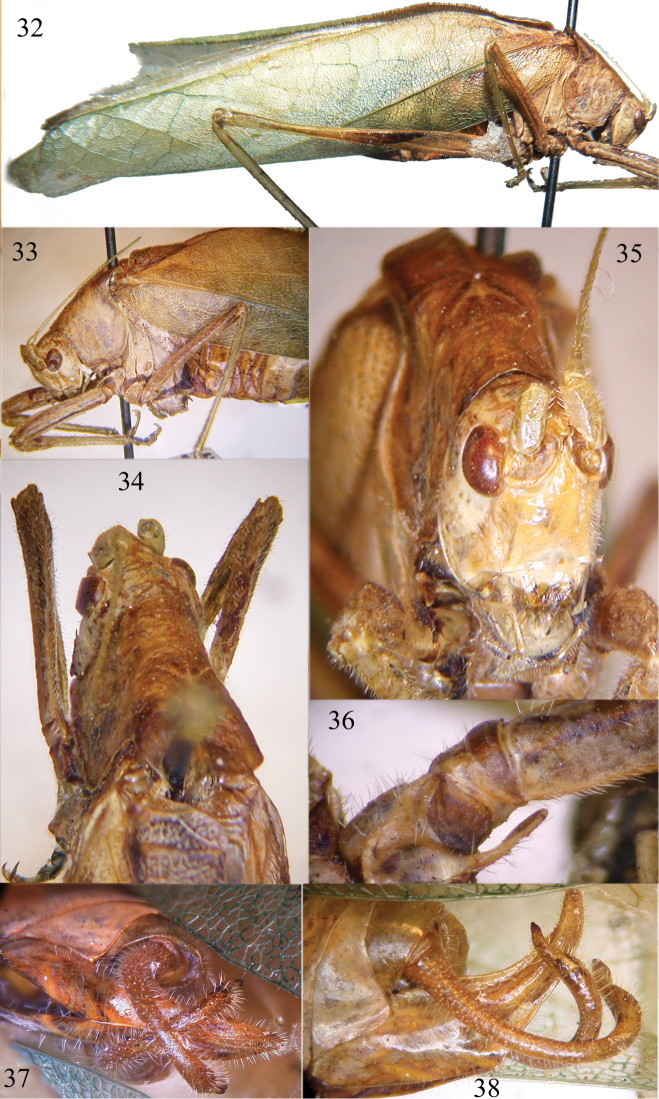
*Angustithorax spiniger* gen. n., sp. n. Lateral view of holotype (**32**), lateral view (**33**) and dorsal view of pronotum (**34**), frontal view of head (**35**), spine on fore coxa (**36**), ventral (**37**) and lateral view (**38**) of cerci and sub-genital plate.

Head and antennae: fastigium of vertex narrow and pointed, not contiguous with the fastigium of frons, much narrower than the first antennal segment (Figs 34–35). Eyes round, moderately prominent (Figs 33–35), placed behind antennae. The scapus is placed within an area with raised margins and is a bit narrower than the eye. Face with fronto-genal carinae below antennae (Fig. 35). Thorax: pronotum as long as high, narrow and compressed, mainly anteriorly, well-developed humeral excision. Legs: upper and lower margins of legs densely covered by hairs. Fore coxae armed with a long and flattened spine (Fig. 36), fore and mid femora laterally compressed (Fig. 34); fore femora with ventral spines, fore tibiae with ventral spines, closed tympanum on inner side and open on outer side. Mid femora and tibiae with ventral spines. Hind femora with ventral spines, hind tibiae with ventral spines. Tegmina well developed, slightly shiny and shorter than hind wings (Fig. 32). Abdomen: male tenth abdominal tergite unmodified, sub-genital plate without styli, very long, upward curved and pointed; cerci are also very long and in-curved, decussate below the sub-genital plate^3^ (Figs 37–38).

##### Diagnosis.

This genus is vaguely similar to *Miltinobates* Sjöstedt, 1902, mainly in the shape of fastigium of vertex and in the sub-genital plate; however, *Miltinobates* has longer and rounded legs, the medial field of fore wings has clear parallel veins, its sub-genital plate is wider and its size is much bigger.

##### Etymology.

From Latin: *angustus* = narrow, *thorax* = cuirass; because of its very slender pronotum and the entire slender habitus.

#### Angustithorax spiniger sp. n.

Taxon classificationAnimaliaOrthopteraPhaneropteridae

http://zoobank.org/73B54715-EB4A-4AF6-9069-AA85F82DCBB3

Figs 32–38

##### Material examined and depository.

Democratic Republic of Congo, Lubumbashi (11°42'1.06"S, 27°31'57.07"E) 3.II.1968 (♂ holotype), T. De Stefani (MRT).

##### General habitus and colour.

Yellow-green. The stridulatory area of left tegmen is brownish.

##### Description.

Male. Head and antennae: fastigium of vertex narrow and pointed, not contiguous with the fastigium of frons, much narrower than the scapus, not furrowed above (Figs 34–35). Eyes round, moderately prominent (Figs 33–35), placed behind antennae. The scapus is placed within an area with raised margins and is just narrower than the eye. Face with sparse hairs, narrow with fronto-genal carinae below antennae, forming a small triangular area (Fig. 35). Thorax: pronotum longer than high, without lateral carinae, much narrow and compressed, mainly anteriorly, surface shiny, well developed humeral excision on the lateral lobes (Fig. 33). Anterior margin of pronotum straight, posterior margin rounded, pronotum lobes rounded on posterior margins, sinuous on lower margins. Legs: upper and ventral borders of legs densely covered by hairs (Fig. 35). Fore coxae are armed with a long and flattened spine (Fig. 36), fore and mid femora are laterally compressed (Fig. 34); fore tibiae have conchate tympanum on inner side and open on outer side; fore femora have 6 inner ventral spines and 2 spines on outer margin, fore tibiae have 6 inner and 4 outer spines on ventral margins, plus 1 spur on each side, upper margin is furrowed and apically unarmed; mid femora have 8 spines on outer ventral margin, mid tibiae have 9 outer and 6 inner spines on ventral margins, plus 1 spur on each side, upper margin is apically unarmed; hind femora have 7 outer and 2 inner ventral spines, hind tibiae have many ventral and dorsal spines plus 2 spurs on inner margin and 3 spurs on outer margin. Tegmina are well developed, slightly shiny and shorter than hind wings, medial field has only crossed veinlets, in the rest of tegmina veinlets delimit small hexagons (Fig. 32). The stridulatory area of left tegmen is comparatively long (Fig. 34). Abdomen: male tenth abdominal tergite is unmodified, styli are absent, sub-genital plate is very long, upward curved and pointed. The cerci are also very long and in-curved, decussate and apically pointed with a black tip (Figs 37–38). The sub-genital plate and cerci are covered by hairs.

Female. Unknown.

##### Measurements.

Body length: 23.3; pronotum length: 5.9; pronotum height: 4.9; hind femur: 20.2; tegmina: 37.2.

##### Diagnosis.

Very slender body, fore coxae are armed with a long and flattened spine, cerci long and decussate.

##### Etymology.

From Latin: *spiniger* = thorny, after the long and stout spine on the fore coxae.

##### Distribution.

Only known from the type locality: Lubumbashi (Democratic Republic of Congo).

### Tribe Odonturini Brunner von Wattenwyl, 1878

#### Genus Atlasacris

Taxon classificationAnimaliaOrthopteraPhaneropteridae

Rehn, 1914

##### Remarks.

The genus is characterized by fastigium of vertex compressed, narrower than first antennal segment, eyes circular, prominent, pronotum markedly selliform with posterior part of lateral lobes strongly inflated, surface smooth and matt, fore coxae may be armed with a small spine or not, femora unarmed, fore tibiae with open tympana on each side, fore wings reduced to short lobes, hind wings vestigial. The male mid tibiae have a much enlarged and up-curved ventral spur, its first tarsal segment is greatly enlarged, more than twice as that of fore legs, the female has not the inflated pronotum and is lacking spurs on mid tarsi (Rehn 1914, Ragge 1980).

#### Atlasacris peculiaris

Taxon classificationAnimaliaOrthopteraPhaneropteridae

Rehn, 1914

**Figures 39–44.**
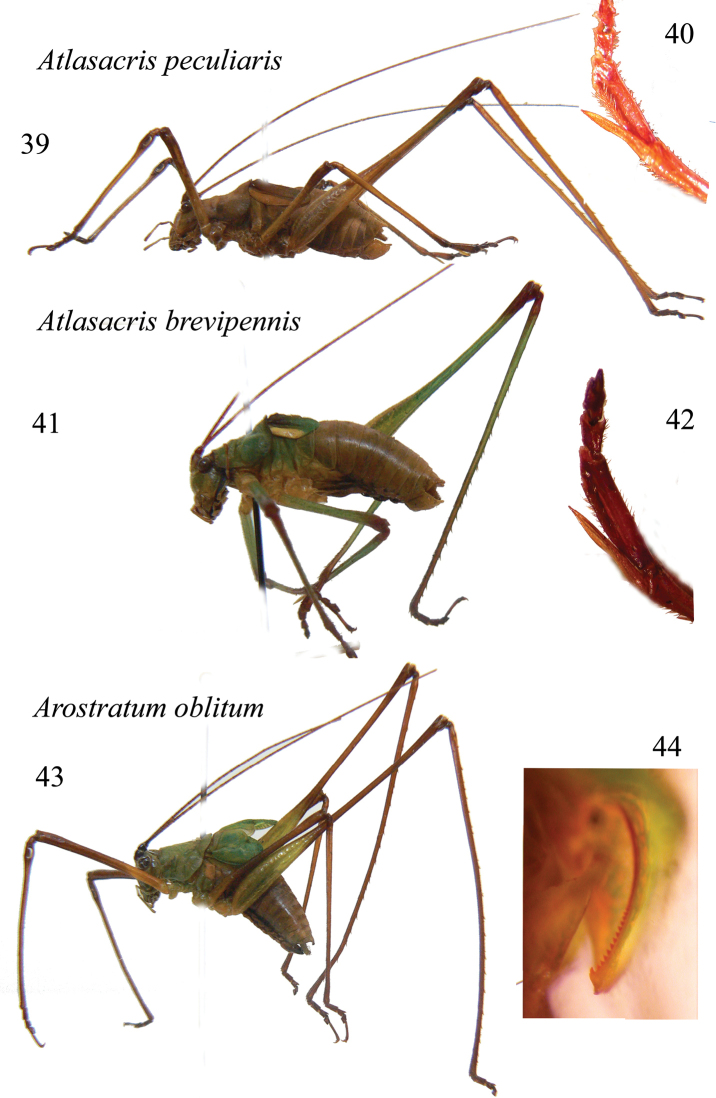
*Atlasacris* and *Arostratum* gen. n. Lateral view of males of *Atlasacris peculiaris* (**39**), *Atlasacris brevipennis* sp. n. (**41**) and *Arostratum oblitum* gen. n., sp. n. (**43**); mid tibia spur of male of *Atlasacris peculiaris* (**40**) and *Atlasacris brevipennis* (**42**); stridulatory file of *Arostratum oblitum* gen. n., sp. n. (**44**).

Figs 39 , 40 , 51 , 53

##### Material examined.

Uganda, Ruwenzori, 2500 m II.1908 (Deutsche Zentr.-Afr.-Exp.), R.Grauer (♂ holotype, originally in alcohol, now mounted); Democratic Republic of Congo, Kwidschwi-Inseln, Kiwu-See, IX.1907 (Deutsche Zentr.-Afr.-Exp) (♂ paratype, originally in alcohol, now mounted) (MfN).

##### Remarks.

The male of *Atlasacris peculiaris* has tegmina just exceeding the 2^nd^ abdominal segment, overlapped for the half of their length, brown with a longitudinal clearer stripe on the fore border (Fig. 39). The most peculiar character is found on the mid tibiae, which end with a long spur, which is little shorter than the first tarsal segment, and is as long as the following ones together (Fig. 40). The female does not show these characters and the tegmina are very short, less than half the pronotum length.

##### Distribution.

According to Ragge (1980) known distribution of *Atlasacris peculiaris* covers the region of Albert-Edward-Kiwa Valley, from Ruwenzori to Burundi.

#### Atlasacris brevipennis sp. n.

Taxon classificationAnimaliaOrthopteraPhaneropteridae

http://zoobank.org/A8212038-F75F-4E48-9A20-CC1B95F27926

Figs 41 , 42 , 46 , 47 , 52

##### Material examined and depository.

NW Tanganyika (now Tanzania) 1910 (♂ holotype), Grauer (MfN).

##### General habitus and colour.

Antennae reddish, green on the face, pronotum and apical parts of tegmina; also femora are green, but their apex is reddish; tibiae are green with apex and base reddish, tarsi are reddish; fore area of tegmina brown, their fore borders cream; abdomen yellow.

##### Description.

Male. Head and antennae: head long, eyes round, prominent. Fastigium of vertex compressed, narrower than the first antennal segment, sulcate above, a small concave in lateral view. Thorax: pronotum without carinae, undulated and selliform, with inflated posterior lateral and hind parts, anterior margin rounded, posterior margin undulate and inflated (Figs 41, 46–47). Legs: the spine on fore coxae is not present, fore tibiae have 3 spines on the outer ventral margin plus apical spur, 2 spines on outer upper margin plus apical spur, and only apical spur on upper inner margin; femora are unarmed. Tympana of fore tibiae are open. Mid tibiae end with a long inner spur and the first tarsal segment is longer than the other ones and shows a long concavity, where the long spur may be hidden (Fig. 42). Tegmina are reduced, just surpassing the 1^st^ abdominal segment, overlapping for most of their median length (Fig. 46). Alae present, but concealed below tegmina. The stridulatory file corresponds to that described by Hemp et al. (2009) for *Monticolaria* Sjöstedt, 1909; it consists of about 50 teeth. The proximal part contains more teeth (about 35) than the much longer distal part which bears around 20 large, asymmetrical and widely spaced teeth. The right tegmen has a wide triangular speculum that covers ca. ¾ of the tegmen length. Abdomen: tenth tergite enlarged, with a wide concavity and two postero-lateral pointed and up-curved tips (Figs 49–50); the sub-genital plate is long and has apically a v-shaped concavity. Styli are absent, but two small protrusions are present on the lateral tips of the sub-genital plate (Fig. 52). Cerci are stout, in-curved and with an apical in-curved pointed tip.

**Figures 45–48.**
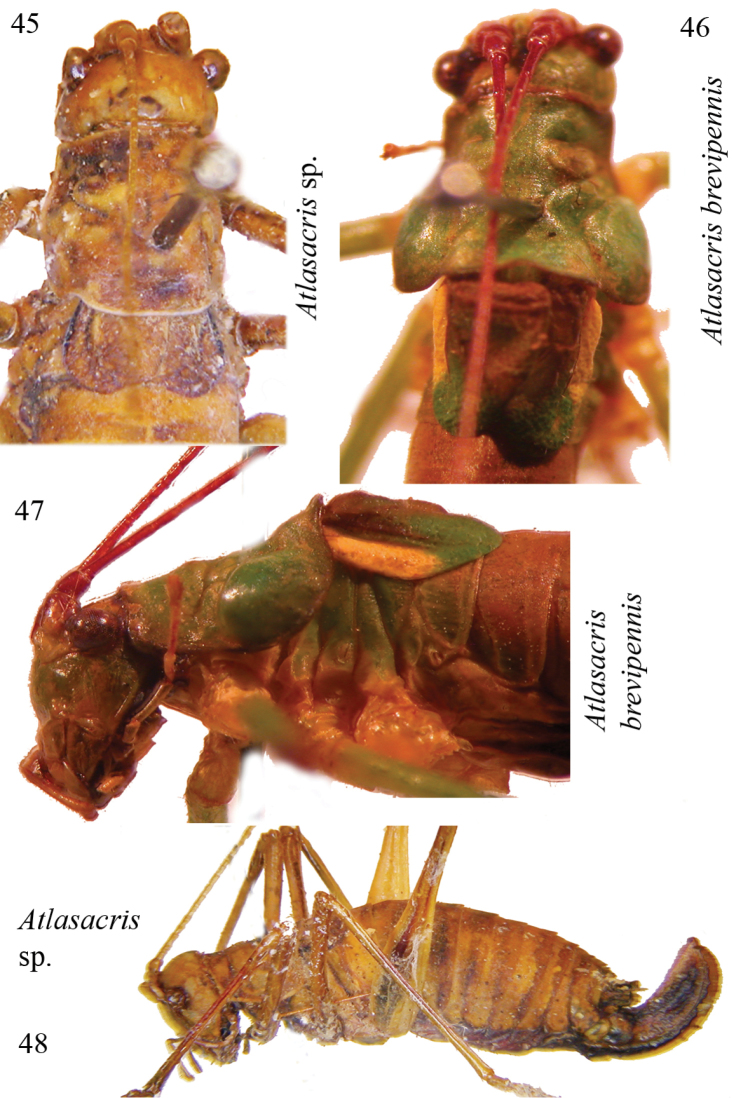
Genus *Atlasacris*. Dorsal view of the female of *Atlasacris* sp. from the Democratic Republic of Congo (**45**), and of the male of *Atlasacris brevipennis* sp. n. (**46**); lateral view of head, pronotum and tegmina of the male of *Atlasacris brevipennis* sp. n. (**47**); lateral view of the female of *Atlasacris* sp. (**48**).

**Figures 49–58.**
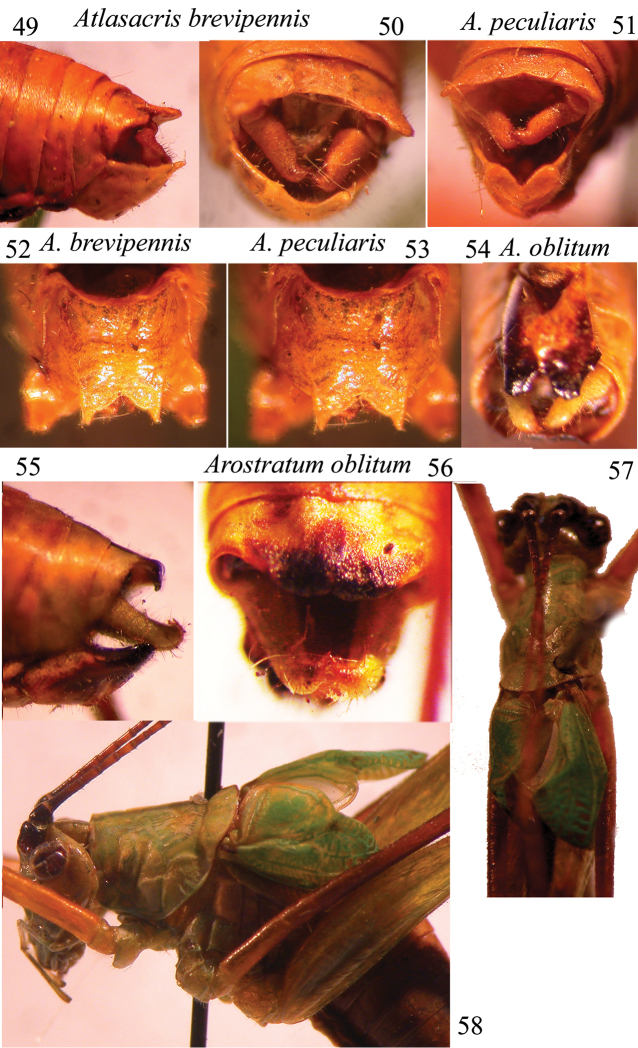
*Atlasacris* and *Arostratum* gen. n. Lateral (**49**) and dorsal (**50**) view of last abdominal segments and cerci of *Atlasacris brevipennis* sp. n.; dorsal view (**51**) of last abdominal segments of *Atlasacris peculiaris*; sub-genital plate of *Atlasacris brevipennis* sp. n. (**52**), *Atlasacris peculiaris* (**53**) and *Arostratum oblitum* gen. n., sp. n. (**54**); lateral (**55**) and dorsal view (**56**) of last abdominal segments of *Arostratum oblitum* gen. n., sp. n.; dorsal (**57**) and lateral (**58**) view of head, pronotum and tegmina of *Arostratum oblitum* gen. n., sp. n.

Female. Unknown.

##### Measurements.

Body length: 16.2; pronotum length: 4.0; fore femur: 7.2; mid femur: 7.4; hind femur: 18.7; tegmina: 3.4.

##### Etymology.

From Latin: *brevis* = short, *pennis* = feather, because of its reduced tegminal lobes.

##### Diagnosis.

*Atlasacris brevipennis* is smaller than *Atlasacris peculiaris* (body length 16.2 vs 17–19.5; pronotum length 4.0 vs 4.6–5.2; tegmina: 4.0 vs 5.2–5.5; hind femora: 18.7 vs 19.5–21.5) and the shape of tegmina is clearly different (Figs 39, 41, 46, 47); speculum of *Atlasacris peculiaris* covers ca. half the length of right tegmen. Tenth tergite, cerci and sub-genital plate of *Atlasacris peculiaris* (Figs 51, 53) are very similar to those of *Atlasacris brevipennis* sp. n.

##### Distribution.

*Atlasacris brevipennis* sp. n. is known only from the type locality: NW Tanzania.

#### Atlasacris sp.

Taxon classificationAnimaliaOrthopteraPhaneropteridae

Figs 45 , 48

##### Material examined.

Democratic Republic of Congo, Kahuzi National Park (1°55'44.37"S, 28°0'50.52"E) (2200–2700 m) 15.IX.1971, T. De Stefani (1♀) (MRT).

##### Remarks.

In the female of *Atlasacris peculiaris* pronotum is not selliform and inflated, mid tibiae do not have the *Atlasacris* characteristic ventral spur and a first long tarsal segment, and the tegmina are very short (♂ 4.6–5.2, ♀ 2.2), less than half the pronotum length (cf. Rehn 1914). The ♀ listed above, characterized by its very short tegmina (1.4 mm) (Figs 45, 48), has a very small spine on the fore coxae, that consents to exclude that it belongs to the gen. *Monticolaria* Sjöstedt, 1910 (Hemp et al. 2009). Due to the existence of another species of *Atlasacris*, described above, the finding of one female without the male does not allow a reliable identification. Here the measurements of the specimen are reported and in parenthesis those of the female paratype of *Atlasacris peculiaris* recorded by Rehn (1914) are given. However, this paratype is no more present in the MfN.

##### Measurements.

Body length: 17.1; pronotum length: 3.6 (4.2); pronotum height: 2.6 (3.5); fore femur: 6.7 (8.0); mid femur: 6.8 (8.0); hind femur: 15.7 (19.0); tegmina: 1.4 (2.2); ovipositor: 6.1 (7.2).

#### Genus Arostratum gen. n.

Taxon classificationAnimaliaOrthopteraPhaneropteridae

http://zoobank.org/66430012-FB2B-4683-B5F1-4D68CA51FDA0

##### Type species.

*Arostratum oblitum* sp. n., here designated.

##### Description.

Head and antennae: 1^st^ antennal segment larger than fastigium, eyes round. Legs: open tympana on both sides of fore tibiae. Coxae unarmed. 4 spines are present on ventral outer margin of fore tibiae, spines on femora are lacking. Mid tibiae without apical spur and first tarsal segment is of normal size. Thorax: pronotum with a small inflated area on metanotum. Tegmina very short, 2^nd^ pairs of wings very reduced. Abdomen: tenth tergite almost straight, cerci in-curved, ending with a pointed tip at right angle. Sub-genital plate with a v-shaped concavity and thickened margins. Styli absent.

##### Diagnosis.

Peculiar characters of this genus are the very long legs, hind femurs being 1.4 longer than the body length. *Arostratum* gen. n. is clearly related to *Atlasacris*, *Monticolaria*, *Odonturoides* and *Meruterrana* Sjöstedt, 1912. *Arostratum* gen. n. shows very unique characters, as the absence of the enlarged spur on the mid tibiae of the male (that are present in males of *Atlasacris*, *Monticolaria*, *Odonturoides* and *Meruterrana*: Ragge 1980) and the very long legs. In *Atlasacris* male hind femurs are 1.1–1.2 longer than the body length (in the female of *Atlasacris specularis* they are as long as the body, Rehn 1914), in *Monticolaria* the male hind femurs are 0.8–1.0 times as the body length. Also the external genitalia are very characteristic, as given in the description of the new species *Arostratum oblitum*.

##### Etymology.

From Latin: *Arostratum* = without rostrum or spur.

#### Arostratum oblitum sp. n.

Taxon classificationAnimaliaOrthopteraPhaneropteridae

http://zoobank.org/D331F578-2CFC-44BD-8187-91CB8A10FF18

Figs 43 , 44 , 54–58

##### Material examined and depository.

NW Tanganyika (now Tanzania) 1910 (♂ holotype), Grauer (MfN).

##### General habitus and colour.

First antennal segments are black, other reddish, pronotum and tegmina green, abdomen brownish, femora green and yellowish, tibiae reddish. The apex of tenth tergite and of sub-genital plate are black.

##### Description.

Male. Head and antennae: fastigium of vertex compressed, much narrower than the first antennal segment, eyes round, prominent (Figs 57, 58). Antennae longer than the body. Legs: open tympana are present on both sides of fore tibiae. Coxae unarmed. 4 spines plus 1 apical spur are present on ventral external margin of fore tibiae, 1 spine is present on outer upper margin and no spurs are present on apical upper margins; mid tibiae with 3 spines on outer ventral margin plus 2 small apical spines; 2 apical spines are also present on inner ventral and on inner dorsal margins; hind tibiae have some spines on ventral margins plus 4 apical spines; many spines are present on upper margins of hind tibiae plus 2 apical ones; spines are absent on femora. Thorax: pronotum without lateral carinae, anteriorly narrower than on the posterior part, similar to that of *Atlasacris*, with a small inflated area on lower and posterior areas (Figs 43, 58). Tegmina are very short, not exceeding the 2^nd^ abdominal segment (Figs 57–58). Wings are reduced to very small scales. As in *Atlasacris*, the stridulatory file matches the model described by Hemp et al. (2009) for *Monticolaria*; it consists of about 50–60 teeth. The proximal part contains more teeth (about 40) than the distal part, which bears around 17–18 large, asymmetrical and widely spaced teeth, in the same space of former 40 teeth. The last tooth on the posterior border of left tegmen is similar to a hook, longer and bigger than previous ones (Fig. 44); it probably produces a sound similar to a click. The right tegmen has a wide triangular speculum that covers ca. ¾ of the tegmen length. Abdomen: tenth tergite almost straight with undulate and down-curved posterior margin (Figs 54, 55, 56), cerci are stout, in-curved, ending with a pointed black tip, placed at right angle (Fig. 56). The sub-genital plate is long with an apical v-shaped concavity and thickened lateral margins. Styli are absent (Fig. 54).

Female. Unknown.

##### Diagnosis.

Small species with very short wings and very long legs, pronotum anteriorly narrower than on the posterior part, with a small inflated area on lower and posterior areas.

##### Measurements.

Body length: 14.2; pronotum length: 3.4; fore femur: 11.2; mid femur: 10.4; hind femur: 20.0; hind tibiae: 24.0; tegmina: 4.8.

##### Etymology.

From Latin: *oblitum* = forgotten. The specimen here treated of *Arostratum oblitum* sp. n. was collected in 1910 and was forgotten for 73 years, when in 1983 D. Ragge studied it and established that it was belonging to one unidentified genus; finally, 104 years after its collection it is described.

### Diagnosis of *Atlasacris*, *Arostratum* gen. n. and related genera

Hemp et al. (2009) have pointed out some characteristics of *Monticolaria*, shared with *Meruterrana*, *Odonturoides* and *Atlasacris*, in particular the presence of a long apical spur on the mid tibiae of males. They share also reduced wings (but *Meruterrana elegans* Sjöstedt, 1912 has less reduced wings). In addition, Hemp et al. (2009) have found in *Monticolaria* a peculiar stridulatory file, consisting of two parts, the distal one composed of large, asymmetrical and widely spaced teeth. The study of the male specimens of *Odonturoides jagoi* Ragge, 1980 (MfN) showed that also in the genus *Odonturoides* Ragge, 1980 the stridulatory file is similar to that found in *Monticolaria*. Moreover, Naskrecki and Bazelet (2011) found a similar stridulatory file in the genus *Austrodontura* Fontana et Buzzetti, 2004. Additionally, the study of stridulatory files in different species of the Palaearctic genus *Odontura* Rambur, 1838 (BMCP) confirmed the same type discovered by Hemp et al. (2009). Thus, the same model was found in African genera *Monticolaria*, *Atlasacris*, *Austrodontura*, *Odonturoides*, *Arostratum* gen. n., and in the Palaearctic genus *Odontura*; however, *Odontura*, *Austrodontura* and *Arostratum* gen. n. do not have the enlarged apical spurs on the mid tibiae in males and the stridulatory file of *Arostratum* gen. n. has a final hook, not present in related genera. *Arostratum* gen. n. probably presents the ancestral state of Phaneropterinae with normal spurs on the mid tibiae and long legs and probably the distal “hook” on the stridulatory file is an apomorphism. Altogether, the similar type of the stridulatory file (that is responsible for the song type and therefore sexual relationships) suggests that also *Arostratum* gen. n. is closely related to *Monticolaria*, *Meruterrana*, *Odonturoides*, *Atlasacris* and *Odontura*.

The genera treated above (except *Odontura*) are located in a region covering Uganda, Kenya, Tanzania, the Democratic Republic of Congo and Burundi, corresponding to the Eastern Arcs. According to Hemp et al. (2009) this area is a well-known hotspot of biodiversity: many of Saltatoria species are endemic to these mountain ranges. The fact that they are sympatric suggests a comparatively old divergence from isolated ancestors on single mountains or single mountainous habitats in respective areas.

## Supplementary Material

XML Treatment for Pardalota asymmetrica

XML Treatment for Diogena denticulata

XML Treatment for Diogena fausta

XML Treatment for Symmetropleura

XML Treatment for Symmetrokarschia

XML Treatment for Symmetrokarschia africana

XML Treatment for Symmetroraggea

XML Treatment for Symmetroraggea dirempta

XML Treatment for Plangiopsis adeps

XML Treatment for Poreuomena sanghensis

XML Treatment for Melidia

XML Treatment for Melidia claudiae

XML Treatment for Tylopsis continua

XML Treatment for Angustithorax

XML Treatment for Angustithorax spiniger

XML Treatment for Atlasacris

XML Treatment for Atlasacris peculiaris

XML Treatment for Atlasacris brevipennis

XML Treatment for Atlasacris sp.

XML Treatment for Arostratum

XML Treatment for Arostratum oblitum

## Footnotes

1 The name *Cameronia* of Bourguignat was overlooked and another genus *Cameronia* was described in 1948 by Basin for a nematode Oxyurida, parasite of *Gryllotalpa*, pre-occupied as well, but currently used (R. Poggi, pers. comm.).

2 Titillators have been recently found in other genera of African Phaneropterinae (Massa 2013).

3 This may be artificial in the mounted specimen and the living insect might hold the cerci otherwise.
